# Where the Most Private Becomes Public: Policy Making for Sexual Health

**DOI:** 10.1371/journal.pmed.1000082

**Published:** 2009-05-26

**Authors:** The PLoS Medicine Editors

Worldwide, the World Health Organization estimated that in 1999 there were 340 million new cases of curable sexually transmitted diseases (STDs)—syphilis, gonorrhea, chlamydia, and trichomoniasis—in men and women aged 15–49 years [1]. Although in Western countries curable STDs may not seem a major threat to public health, these diseases disproportionately affect the poor, young people, and ethnic minorities, and can cause acute illness, disability and death, pre-term or low birth-weight babies, congenital birth defects, female infertility, and increased HIV transmission. High-income countries are by no means exempt from the burden of STDs: there are 19 million new cases of STDs each year in the United States, at an estimated cost of US$15.9 billion annually to the US health care system [2].

Yet the burden of morbidity and mortality from STDs is only one aspect of sexual health. Formerly considered under the umbrella of reproductive health by policy makers, sexual health was identified as a topic meriting attention in its own right by the World Health Organization in 2004 [3]. The International Planned Parenthood Foundation (http://www.ippf.org/en/), a leading advocate of sexual and reproductive health and rights for all, has endorsed the United Nations definition of sexual health: “the notion of sexual health implies a positive approach to human sexuality and the purpose of sexual health care should be the enhancement of life and personal relations and not merely counselling and care related to reproduction and sexually transmitted diseases.”

The International Planned Parenthood Foundation has identified five priority areas for action on sexual health: addressing unsafe abortion, access to services and information for marginalized individuals, access to contraception, advocacy for better legislation and services, and action on HIV/AIDS (which is not categorized as a curable STD). Tackling such priority areas, and reducing the burden of illness and death that results from sexual health problems, requires approaches that are tailored to the needs of different individuals and groups in different countries. Since our launch in 2004, *PLoS Medicine* has highlighted some of these diverse approaches.

Unmet contraceptive needs and unsafe sex both figure in the top 20 risk factors for mortality and burden of disease [4]–[6] and are included among *PLoS Medicine*'s recently announced priority areas for publication [7]. Several of the UN Millennium Development Goals (MDGs) are relevant to these problems—in particular, MDG3 (promoting gender equality and empowering women), MDG5 (improving maternal health), and MDG6 (combating HIV and other diseases). A report published in 2008 [8] details some of the progress that has been made towards achieving the aims of MDG5. One target of MDG5 is to achieve universal access to reproductive health care by 2015. Although some progress has been made, the report noted that 20% of sub-Saharan African women and 27% of poor women from Latin America and the Caribbean have unmet contraceptive needs. Lack of access to contraceptives results in unnecessary maternal deaths; complications from unsafe abortion account for about 70,000 deaths each year.

Access to free or affordable contraceptives remains limited, even in many high-income countries. For example, in the US, health insurance companies in many states do not cover the costs of contraception. Moreover, according to the Center for Reproductive Rights (http://reproductiverights.org/en/project/contraceptive-access-in-the-united-states) “funding for the U.S. government's Title X program, which funds low-cost, confidential family planning services, is 61% lower today in constant dollars than it was in 1980.” Change may be on the way: the Obama administration has now moved away from “abstinence only” education favored by the Bush administration as a panacea for preventing unwanted pregnancy and STDs, and has lifted a ban on federal funding for foreign family planning agencies that promote or provide information about abortion [9]. We recently published a study and accompanying commentary [10],[11] that highlighted the potential benefits of “abstinence-plus” programs, which promote abstinence to reduce HIV transmission but also promote safe sex.

By contrast, despite the ready availability of free contraceptives in the United Kingdom, pregnancy rates (intended and unintended) are high in teenage girls [12]. Such teenage pregnancies are associated with low birth-weight babies, and can result in significant disadvantages for mothers and their children. The high rate of teenage pregnancies has led to initiatives such as advertising condoms more prominently on TV at times that under-18s are likely to be viewing. New studies are clearly needed to unravel the complex reasons underlying the high rate of STDs and teenage pregnancies, and to define effective strategies to tackle this problem. Judith Stephenson and colleagues, for example, recently examined the impact of a peer-led sex education program in the UK [13] and concluded that although this innovative approach didn't result in a reduction in abortions, it may have led to slightly fewer live births among the teenagers. Lee Warner and colleagues assessed the effect of a brief video screened in the waiting room of STD clinics in the US and found that this simple intervention reduced new infections by almost 10% overall in three clinics [14]. Both studies highlight the complexity of issues surrounding improving sexual health and promoting safe sex, but also suggest that innovative approaches aimed at modifying sexual risk behavior may improve health outcomes.

Addressing the morbidity and mortality that results from unsafe sex requires pinpointing who is at risk and why. For instance, while resources are earmarked to ensure the routine availability of testing for HIV and other STDs in some groups, such as pregnant women in the developed world, these infections are on the rise in other groups whose needs are currently not being addressed, such as those old enough to have stopped worrying about unwanted pregnancy. The UK Health Protection Agency reports that STDs other than HIV have doubled in less than a decade in those aged over 45 years [15]. In Brazil, HIV is an increasing problem in those aged over 50 years, with cases doubling from 7.5 to 15.7 cases per 100,000 inhabitants between 1996 and 2006 [16]. Clearly there is a need to more effectively target older individuals to promote healthy sexual behavior. In the developing world, complex relationships have also been found between behavior and the risk of unsafe sex. For example, Sheri Weiser and colleagues found that food insufficiency among women in Botswana and Swaziland is associated with an increased risk in women of unsafe sex [17],[18]. Alcohol abuse has also been linked to a risk of unsafe sex and HIV infection in men and women in Botswana [19]. Tackling HIV thus remains a multifaceted challenge that requires attention to issues well beyond screening for and treatment of infection.

Promoting sexual health requires both systematically integrated and individually tailored approaches. Initiatives must reach out in new ways to target those who are at risk. Although narrowly focused political and religious perspectives have in the past hampered policy making to improve sexual health, today's politicians and religious leaders must redouble their leadership in tackling these problems, precisely because they occur at the intersection of health, culture, religion, and politics. Only in the setting of such support can medical research fulfill its role in promoting sexual health, be it through the use of new media, as reported by recent *PLoS Medicine* papers exploring the utility of the Internet for reducing the incidence of STDs among specific groups in the developed and developing world [20]–[22], or through more traditional studies examining how new drugs, or novel educational packages, can be deployed effectively. It is time to realign research and policy making to promote better sexual health for all.

## Abbreviations

MDG: Millennium Development Goal
STD: sexually transmitted disease
